# Genetically Engineered Mouse Models of Brain Cancer and the Promise of Preclinical Testing

**DOI:** 10.1111/j.1750-3639.2008.00234.x

**Published:** 2009-01

**Authors:** Jason T Huse, Eric C Holland

**Affiliations:** Department of Cancer Biology and Genetics, Memorial Sloan-Kettering Cancer CenterNew York, N.Y.

**Keywords:** glioma, medulloblastoma, clinical trials, targeted therapy, murine model

## Abstract

Recent improvements in the understanding of brain tumor biology have opened the door to a number of rational therapeutic strategies targeting distinct oncogenic pathways. The successful translation of such “designer drugs” to clinical application depends heavily on effective and expeditious screening methods in relevant disease models. By recapitulating both the underlying genetics and the characteristic tumor-stroma microenvironment of brain cancer, genetically engineered mouse models (GEMMs) may offer distinct advantages over cell culture and xenograft systems in the preclinical testing of promising therapies. This review focuses on recently developed GEMMs for both glioma and medulloblastoma, and discusses their potential use in preclinical trials. Examples showcasing the use of GEMMs in the testing of molecularly targeted therapeutics are given, and relevant topics, such as stem cell biology, *in vivo* imaging technology and radiotherapy, are also addressed.

## INTRODUCTION

The treatment challenges posed by primary brain tumors are manifold and are enhanced by unique features of the intracranial environment. For instance, the well-established ability of many brain tumors to widely diffuse into surrounding normal parenchyma frequently precludes complete surgical resection. Additionally, pharmaceutical intervention can be hampered by the blood–brain barrier and the inability to effectively deliver drugs into a given tumor mass. The development of effective therapeutics is further complicated by the genetic heterogeneity exhibited not only between tumors of the same subtype, but also within individual tumors. Recent work has highlighted such molecular complexity in both glioma and medulloblastoma, the most common primary brain tumors in adult and pediatric populations, respectively (20, 67). Nevertheless, the mainstays of nonsurgical treatment for both of these conditions remain strikingly similar, consisting of some combination of radiation and cytotoxic chemotherapy (67, 86). For glioblastoma multiforme (GBM), the most malignant variant of glioma, the alkylating agent temozolomide in combination with radiotherapy has recently resulted in a marginal increase in median survival to approximately 15 months (74), and now represents the standard of care for a variety of glioma subtypes. Radiation and chemotherapy have shown greater efficacy in the treatment of medulloblastoma, where 5-year survival rates are now as high as 70%–80% (21). However, the long-term side effects of these conventional modalities, especially when applied to the developing brain, remain problematic.

The shortcomings of the current standards of care highlighted above underscore the pressing need for rationally conceived therapies targeted to the specific molecular pathways deregulated in primary brain tumors. Recent advances in tumor biology have revealed no shortage of potential targets for therapeutic intervention, and new compounds are being developed continually. The obvious next challenge has now become the efficient screening of promising therapies, either alone or in combination, in biologically relevant systems. In clinical oncology, most drugs fail in late development after enormous financial investments, typically due to a lack of efficacy in human subjects. This places a premium on quality preclinical testing to (i) select appropriate molecular targets; (ii) determine the effectiveness of drugs directed against those targets and the ideal genetic and cellular context for their use; (iii) evaluate the toxicity of selected drugs; and (iv) identify relevant biomarkers demonstrating drug efficacy and specificity to assist in subsequent clinical trials (71).

Cancer modeling for preclinical testing relies on both *in vitro* and *in vivo* systems. Tumor-derived cell lines play an important role in this process and are often the initial reagents employed for drug screening because of their ready availability and ease of use. However, the inability of cell culture experiments to fully recapitulate both the genetic and cellular heterogeneity of tumors and the complexity of tumor-stroma interactions places obvious limitations on the extent to which data derived from such studies can be interpreted. Xenograft analysis represents the most frequently used *in vivo* modeling system for the testing of anticancer therapeutics, primarily because of low cost and ease of implementation. To form a xenograft, primary tumor cells or cell lines are injected either subcutaneously or orthotopically (into the native tumor site) into immunocompetent or immunonaive mice. The shortcomings of this approach as a high-fidelity cancer model center both on the inability of extensively passaged cell lines to accurately represent the diverse molecular and cellular characteristics of naïve tumors and the failure of foreign transplantation sites to fully embody the native stromal microenvironment (5, 16, 71). While these problems can be addressed somewhat by the use of minimally passaged tumor cells and exclusively orthotopic transplantation, issues concerning the perturbed stromal setting of immunodeficient murine hosts remain. Therefore, it seems hardly surprising that xenograft testing for cancer drug development has demonstrated limited predictive value (71).

The recent development of several distinct murine models of medulloblastoma and glioma (both astrocytic and oligodendroglial variants) has provided more physiologically relevant *in vivo* systems for the evaluation of anticancer therapies. While genetically engineered mouse models (GEMMs) also have their limitations (see below), they more accurately recapitulate the casual genetic events and subsequent molecular evolution of brain tumors as they form *in situ*. Furthermore, some GEMMs give rise to tumor-stroma interactions resembling those found in native tumors, and appear to harbor cellular subpopulations like cancer stem cells (CSCs) thought to be of central importance to the development, maintenance and drug resistance of brain cancer. This review will summarize the current state of genetically engineered mouse modeling for both glioma and medulloblastoma in the context of developing more effective preclinical screening methods for novel cancer therapeutics.

## GEMMS AS MODELS FOR PRECLINICAL TESTING

As highlighted elsewhere in this issue, GEMMs provide powerful systems with which to address many of the pressing issues in modern cancer biology, including but not limited to the molecular mechanisms and cellular origins of neoplastic processes, along with the importance of the tumor microenvironment. Nevertheless, not all GEMMs are suitable for preclinical testing for any one of a number of factors. The ideal mouse model for drug development would (i) faithfully recapitulate the genetics and molecular characteristics of the human tumor in question; (ii) posses a short tumor latency and high penetrance; (iii) be relatively simple to generate and easy to use; and (iv) ideally, would contain a built-in mechanism to assess therapeutic effects, such as a bioluminescent reporter (see below). In reality, however, these criteria frequently position themselves at odds with each other, making it difficult to completely satisfy them. For instance, models characterized by rapidly forming tumors may not be sufficiently representative of their human counterparts, especially with regard to issues of the evolving microenvironment and the impact of additional stochastic genetic events. GEMMs with short tumor latency may also contain oncogenic drivers irrelevant to the human tumor in question and express them so diffusely as to cause multifocal lesions, a situation more reminiscent of cancer-predisposing syndromes than of conventional unifocal tumorigenesis. Conversely, a single-minded focus on precisely recapitulating all aspects of a given human cancer for the purpose of drug testing has significant drawbacks as well. Such an approach not infrequently leads to complex models characterized by sophisticated genetic engineering that while biologically informative, may be challenging to effectively implement in therapeutic trials.

On a related note, recent advances in genomics have underscored the molecular heterogeneity exhibited by most brain tumors (2, 20, 22, 56, 67) and further emphasized that even individual diagnostic categories are at best collections of genetically overlapping yet distinct disorders that cannot be effectively represented by a single GEMM. A more effective approach would seemingly be to employ multiple GEMMs, each driven by relevant genetic abnormalities that together encompass the full spectrum of molecular variability inherent in these neoplasms. In this way, even relatively simple models, driven by perturbations in single oncogenic pathways, could be of considerable use, especially when testing drugs targeting those specific pathways. None of the GEMMs described in the ensuing sections represents a perfect model for either glioma or medulloblastoma with regard to preclinical trials. However, many strike a workable balance between the criteria discussed above, and, when taken together, embody a powerful resource for the testing of promising treatment strategies.

## MOUSE MODELS OF GLIOMA

### Molecular pathology

**Figure 1:**
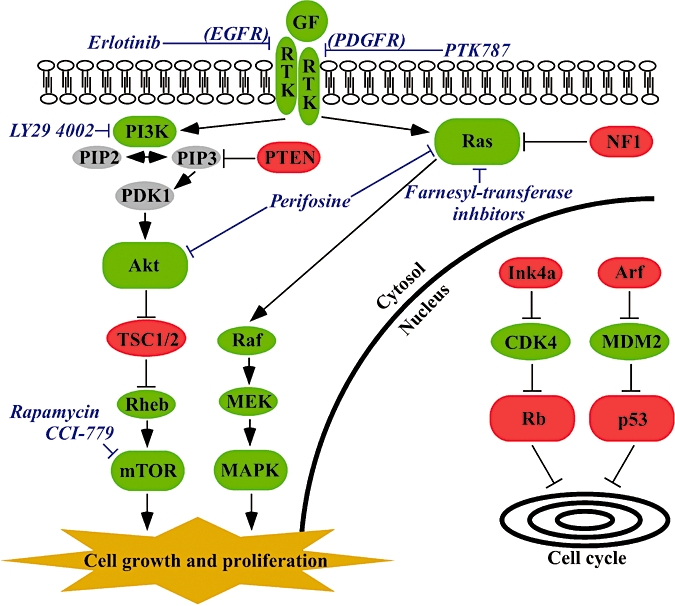
Glioma-implicated signaling pathways that have been employed in the production of genetically engineered mouse models. Oncogenes are shown in green, and tumor suppressors are shown in red. Examples of pharmaceutical agents are shown in italicized blue with their targets indicated.

Over the past two decades, investigations into the pathogenesis of the various glioma subtypes have revealed central roles for a defined set of key biological pathways (Figure 1). The molecular pathology of glioma has been reviewed extensively elsewhere (2, 20, 37, 51); this article will attempt to highlight important features as they relate to recent successes in the murine modeling of primary astrocytic and oligodendroglial tumors. In light of this, it is worth mentioning that the genetic abnormality most frequently associated with oligodendroglioma, namely loss of chromosome 1p and 19q (62), has not been effectively modeled in mice, partly because the precise genes involved are not yet known.

The disruption of a set of tumor suppressor pathways with direct effects on cell cycle control appears be crucial in the evolution of glioma. The *p53* gene is either mutated or deleted frequently in astrocytic gliomas, particularly those that progress from low-grade astrocytoma to GBM (so-called secondary GBM) (10, 45, 53). Additionally, retinoblastoma (*RB*) is mutated in 10%–25% of high-grade astrocytomas, and functionally silenced in another ∼15% through amplification of its antagonist *CDK4*(10, 30, 53). Similar effects on the RB pathway are also frequently seen in anaplastic oligodendroglioma (83). Lastly, the tumor suppressors INK4A and ARF, positive regulators of RB and *p53* respectively, are encoded at the *CDKN2A* locus, which is deleted in approximately 50% of high-grade astrocytomas and a significant percentage of anaplastic oligodendrogliomas as well (8, 10, 53, 77).

Receptor tyrosine kinases (RTKs) and their downstream signaling pathways have been established as the primary oncogenic drivers in multiple glioma subtypes. Amplification of the epidermal growth factor receptor locus (*EGFR*) occurs in ∼40% of GBM, and is considered to be the defining genetic lesion of so-called primary GBM (GBM that arises *de novo* rather than evolving from a lower grade astrocytic lesion) (10, 53, 90). Furthermore, a constitutively active deletion mutant of EGFR, EGFRvIII, is found in 20%–30% of GBM (18). Platelet-derived growth factor (PDGF) and its receptor (PDGFR) are frequently upregulated in both low-grade astrocytoma and oligodendroglioma along with a defined subset of GBM (∼13%), and the elevated expression of both suggests that autocrine/paracrine loops between ligand and receptor enhance their impact in glioma biology (10, 13, 53, 87).

The PI3K/AKT/mTOR pathway, operating downstream of RTKs, exerts profound effects on cell growth, proliferation and metabolism, and has been reported to be activated in up to ∼85% of GBM (10, 53, 82). The phosphatase and tensin homolog (PTEN) constitutes the primary negative regulator of this pathway, and mutation or deletion of the *PTEN* gene, often by way of complete loss of its locus on chromosome 10q, is found in a large percentage of GBM (10, 38, 53). Furthermore, while discrete PTEN mutations are much less frequent in high-grade oligodendroglial lesions, loss of chromosome 10 remains common (8). The RAS/MAPK pathway, also positioned downstream of RTKs, provides an additional mitogenic stimulus that is often dysregulated in astrocytic glioma, despite a notable absence of activating *RAS* mutations in most high-grade variants (10, 24, 53). Loss of the tumor suppressor NF1, a negative regulator of RAS signaling, may be central to this process. Two recent large-scale sequencing projects have identified frequent *NF1* mutations in sporadic GBM (10, 53). Additionally, germline *NF1* mutations define the familial condition of neurofibromatosis 1, whose central nervous system (CNS) manifestations include increased incidence of diffuse atrocytoma, and, most commonly, low-grade glioma of the optic nerve and chiasm (OPG) (19).

### Engineering and characteristics

Several GEMMs with considerable promise for preclinical testing have been developed in recent years using combinations of the glioma-associated genetic lesions highlighted in the preceding paragraphs (Table 1). One such model for astrocytic glioma has been established using combined *Nf1* and *p53* mutants (64). These mice develop tumors spanning a range of histological grades within a reasonable latency period (92% at 6 months), most resulting from loss of heterozygosity at the remaining wild-type *Nf1* and *p53* locus (the genes are located so close to each other in mice that separate recombination events are rare). Furthermore, tumors arising in older mice appear to be higher grade, implying that this GEMM may have relevance in the modeling of more slowly evolving astrocytic lesions like secondary GBM. Other non-CNS cancers, most notably sarcoma, do arise in these mice at significant rates. Nevertheless, astrocytomas appear to be the most frequent tumor type found in two of the four genetic backgrounds tested.

**Table 1:** Murine models of glioma shown with underlying genetics, mechanism of engineering, morphologic characteristics and incidence. Abbreviations: TG = transgenic; KO = knockout; Astro = astrocytic; Oligo = oligodendroglial; HG = high grade; LG = low grade.

Genetics	Mechanism	Morph/grade	Incidence	Reference
*Nf1*+/−*; p53*+/−	Conventional KO	Astro/variable	92% by 6 months	(64)
*Nf1*+/−*; p53*+/−	Conventional and conditional KO (GFAP-Cre)	Astro/variable	100% by 5–10 months	(96)
*Nf1*+/−*; p53*+/−*; Pten*−/−	Conventional and conditional KO (GFAP-Cre)	Astro/HG	100% by 5–8 months	(41)
*GFAPT_121_*	TG	Astro/LG	100% by 10–12 months	(92)
*GFAPT_121_; Pten*−/−	TG; conditional KO (MSCV-Cre)	Astro/HG	100% by 6 months	(93)
*GFAP-V^12^Ras*	TG	Astro/HG	100% by 0.5–3 months	(14)
*GFAP-V^12^Ras; EGFRvIII*	TG; adenovirus	Oligo/HG	100% by 3 months	(15)
*GFAP-V^12^Ras; Pten*−/−	TG; conventional KO	Astro/HG	100% by 6 weeks	(84)
*S100-v-erbB*	TG	Oligo/LG	60% by 12 months	(85)
*S100-v-erbB; Ink4a/Arf*−/−	TG; conventional KO	Oligo/HG	100% by 12 months	(85)
*S100-v-erbB; p53*+/−	TG; conventional KO	Oligo/variable	100% by 12 months	(85)
*PDGF-B*	MoMuLV	Oligo/variable	40% by 10 months	(79)
*kRas; Akt*	RCAS	Astro/variable	25% by 3 months	(32)
*kRas; Pten*−/−	RCAS; conditional KO (RCAS-Cre)	Astro/variable	60% by 3 months	(36)
*kRas; Akt; Ink4a/Arf*−/−	RCAS; conventional KO	Astro/variable	20%–50% by 3 months	(78)
*PDGF-B*	RCAS	Oligo/variable	60%–100% by 3 months	(12, 72)
*PDGF-B; Ink4a/Arf*−/−*; Pten*−/−	RCAS; conventional KO; conditional KO (RCAS-Cre)	Oligo/HG	60%–100% by 3 months	(12)*
*FIG-ROS; Ink4a/Arf*/−	Conditional TG (Adeno-Cre); conventional KO	Astro/variable	100% by 3 months	(11)
*Nf1*+/−*^GFAP^CKO*	Conditional KO (GFAP-Cre) in *Nf1*+/− mice	Optic glioma	100% by 3 months	(3, 4)

* Fomchenko and Holland, unpublished results.

A similar model has been developed more recently by pairing a *p53* mutant allele with an *Nf1* allele flanked by loxP sites (a so-called floxed allele). Crossing these mice with a transgenic line expressing cre-recombinase under the glial fibrillary acidic protein (GFAP) promoter (GFAP-Cre) eliminates *Nf1* in GFAP-expressing astrocytes and glial precursors, effectively generating an *Nf1*/*p53* double mutant in this cellular subpopulation (96). In this way, tumor-initiating loss of heterozygosity is largely limited to the brain. As would be anticipated, the incidence of non-CNS pathology is dramatically reduced in this model. This benefit, however, is balanced by a somewhat longer disease-free latency (20–40 weeks), although complete penetrance remains. The tumors exhibit a primarily astrocytic morphology, and frequently harbor high-grade features, such as microvascular proliferation and pseudopalisading necrosis. Not surprisingly, the addition of a mutant *Pten* allele to this model both decreases latency (to approximately 10–20 weeks) and increases tumor grade (41). As a side note, GFAP-Cre-mediated homozygous deletion of *Nf1* in the absence of *p53* mutagenesis, while unable to produce parenchymal gliomas in mice, generates OPGs with robust penetrance (3, 4, 97).

A separate set of GEMMs incorporating modulation of the Rb pathway have been generated by transgenically expressing a truncated SV40 T antigen (T_121_) under the GFAP promoter. This mechanism effectively inactivates the Rb pathway in mature astrocytes and their precursors, and leads to fibrillary astrocytomas in adult mice (approximately 100% incidence at 300 days) (92). Furthermore, the presence of *Pten* null heterozygosity in this model significantly decreases disease-free latency and appears to enhance tumor grade in terms of cellularity and mitotic activity (92, 93). Although the dependence of this model on the expression of a viral antigen is questionable from the standpoint of pure physiologic relevance, it remains the only glioma GEMM produced to date whose biology is centered on the Rb pathway.

Another large set of glioma GEMMs feature the overexpression of relevant oncogenes—either RTKs or their downstream effectors—coupled frequently with tumor suppressor loss. A series of transgenic lines has been established, largely based on the expression of a constitutively active Ras (V^12^Ras) under the GFAP promoter (14, 15, 70, 84). The basic transgenic, expressing only V^12^Ras, consistently develops astrocytic tumors, whose histological grade, latency, and, at times, multifocality, appear to depend on transgene dosage (14). High levels of V^12^Ras expression, for instance, yield multiple grade IV lesions per mouse arising within the first 2 weeks of life. Despite their strikingly rapid onset, the induced tumors themselves appear to accumulate additional genetic and molecular alterations as they evolve from lower-grade precursors (14, 70). These changes are reminiscent of glioma pathogenesis and include decreased or absent expression of the tumor suppressors *Pten*, *Ink4a* and *Arf*, and overexpression of *Egfr*, the *p53* antagonist *Mdm2*, and the cell cycle regulator, *Cdk4*. Such findings perhaps bolster the physiologic relevance of this mouse model despite its reliance on a mutated Ras protein not characteristic of glioma biology. The additional expression of EGFRvIII in this GEMM, either in a GFAP-driven transgene or by adenoviral-mediated gene transfer, both decreases disease-free latency and increases tumor grade while also inducing oligodendroglial histological features (15, 84). Finally, deletion of *Pten*, as in many other models, decreases the age of tumor onset and potentiates the development of high-grade lesions (84).

Another similar murine model of oligodendroglioma utilizes transgenic expression of a transforming variant of EGFR, v-erbB, under the S100ß promoter (85). The resulting tumors tend to be low grade, with 60% incidence in 12 months. However, this model does not require a constitutively active Ras variant to induce glioma formation. Furthermore, when v-erbB overexpression is paired with *Ink4a/Arf* loss, tumor incidence increases to nearly 100% in 12 months, and high-grade features predominate. Intermediate effects are seen when either *Ink4a/Arf* or *p53* null heterozygotes are used instead.

Many of the GEMMs described thus far achieve their effects largely through the widespread expression of an oncogenic transgene across the brain. Alternatively, tumor suppressors are mutated in an equally extensive geographical distribution. While such strategies have generated histologically and molecularly relevant glioma, and, as we shall see, medulloblastoma models, their field cancerization effects are perhaps more akin to tumor-predisposing conditions, such as Li Fraumeni syndrome and neurofibromatosis than sporadic brain tumorigenesis. The frequent occurrence of multifocal lesions or even fulminant widespread pathology in some of these models perhaps emphasizes this point (14, 92). The use of viruses for more localized gene delivery has emerged as an alternative mechanism for the production of brain tumors in GEMMs. The tight geographical restriction of cancer-forming genetic events provided by viral transduction better resembles the analogous human condition. Additionally, most viral systems allow for the simultaneous delivery of multiple genes of interest, each in a different viral particle, in a variety of combinations, thus offering a fast experimental readout by circumventing the often painstaking germline mutagenesis required to make multiple, distinct transgenic or knockout lines. The drawbacks of this approach mainly concern reduced tumor incidence in some models, and limitations on the size of genes that can be effectively packaged within the viral vectors themselves. Additionally, the actual delivery of the viral reagent to the mouse brain, typically by injection, may represent a technical challenge for some.

**Figure 2:**
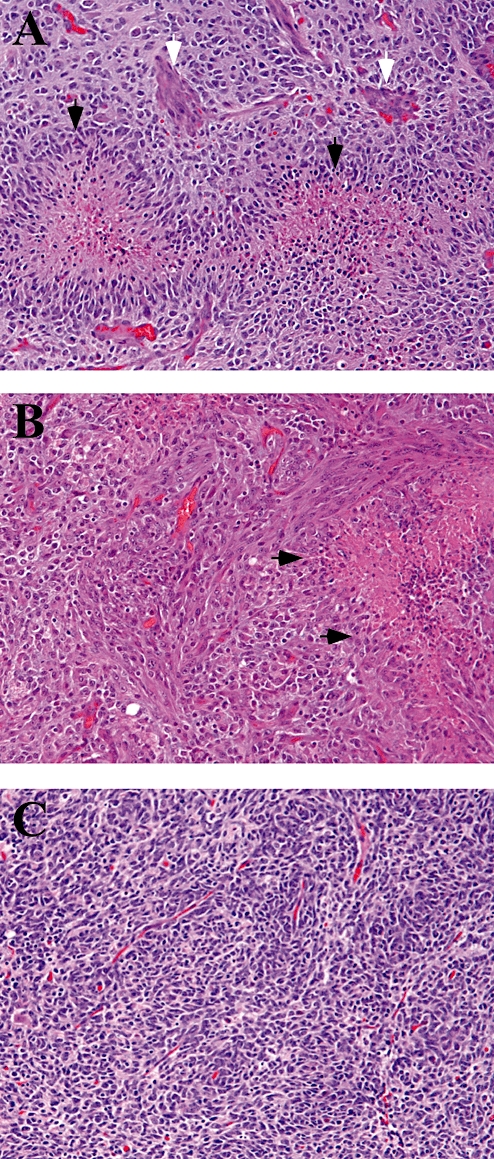
*Examples of murine brain tumor models incorporating RCAS/tv-a technology*. A. High-grade glioma driven by RCAS-PDGF and RCAS-Cre in an *Ntv-a; Ink4a/Arf*−/−; floxed PTEN background. Black arrows indicate pseudopalisading necrosis, and white arrows highlight foci of microvascular proliferation. B. High-grade glioma driven by RCAS-kRAS and RCAS-Akt in an *Ntv-a; Ink4a/Arf*−/− background. Black arrows indicate necrosis. C. Medulloblastoma driven by RCAS-SHH in an Ntv-a background. All micrographs were taken at 20× magnification.

One of the initial workable models of brain cancer using viral expression of a relevant oncogene utilized a murine retrovirus (MoMuLV) to deliver the PDGF B-chain (PDGF-B) into the forebrains of newborn mouse pups (78). Approximately 40% of mice developed tumors, whose histology spanned a wide range, resembling either GBM or primitive neuroectodermal tumor (PNET) for the most part, most likely reflecting heterogeneity in their cells of origin. More recently, a series of GEMMs have been generated employing an avian retrovirus, RCAS, for gene transfer while genetically engineering its receptor, tv-a, into strains of mice under the GFAP or nestin promoters (Gtv-a and Ntv-a, respectively) (32, 33) (Figure 2). In this way, the expression of exogenous transcript is restricted geographically, as well as by cell type, to either astrocytes (Gtv-a) or glioneuronal progenitors (Ntv-a). The RCAS/tv-a model yields astrocytic tumors (∼25% incidence in 12 weeks) in the N-tva background when constitutively active variants of both kRas and *Akt* are used in combination as oncogenic drivers (34), and deletion of *Pten* in these mice appears to phenocopy the effects of RCAS-mediated *Akt* overexpression (36). Tumor incidence and grade are increased when either Ntv-a or Gtv-a mice harboring homozygous *Ink4a/Arf* deletion are used (79). For instance, Ntv-a/*Ink4a/Arf* null mice injected with both RCAS-kRas and RCAS-*Akt* demonstrate near 50% incidence in 12 weeks, with some tumors exhibiting microvascular proliferation and necrosis. The subsequent development of RCAS-PDGF-B vectors has led to further advances in the utility of this model system (12, 72). Ntv-a or Gtv-a mice injected with RCAS-PDGF-B form oligodendroglial or mixed oligoastrocytic tumors at high rates–up to 100% incidence at 12 weeks depending on gene dosage (72). As in other models, loss of tumor suppressors like *Ink4a/Arf* and *Pten* dramatically decreases disease-free latency and increase the appearance of high-grade features (12).

Lastly, another group has developed a virally mediated glioma GEMM driven by a constitutively active fusion RTK (FIG-ROS). While the presence of FIG-ROS in glioma has been seen in only two tumor-derived cell lines to date, overexpression of the native ROS kinase does occur somewhat more frequently in actual tumors (∼30% of cases) and activates similar signaling cascades to those directed by EGFR and PDGFR (91). The model features a floxed stop codon immediately preceding the *FIG-ROS* transgene that is then removed by a Cre-expressing adenovirus allowing transcription (11). When this approach is applied in mice lacking *Ink4a/Arf*, astrocytic tumors form, exhibiting a range of histological grades, most within a reasonable temporal window (∼80% incidence at 15 weeks). Furthermore, activation of relevant signaling networks, such as the *Akt* pathway, is present, perhaps pointing to a more generalized applicability for this model to studies like preclinical testing, despite its reliance on a genetic lesion not associated with the vast majority of gliomas.

## MOUSE MODELS OF MEDULLOBLASTOMA

### Molecular pathology

Multiple molecular pathways and genetic abnormalities have been implicated in the pathogenesis of PNET and its most common variant, medulloblastoma. As for glioma, this review will focus primarily on the molecular mechanisms that have been employed most frequently in the construction of medulloblastoma GEMMs showing promise for preclinical studies. This will unfortunately exclude any discussion of wingless and ERBB signaling, both of which appear to play a central role in medulloblastoma, as well as the most frequent genetic lesion associated with the tumor, isochromosome 17q. For more extensive coverage of these subjects, readers are encouraged to look elsewhere (21, 67).

**Figure 3:**
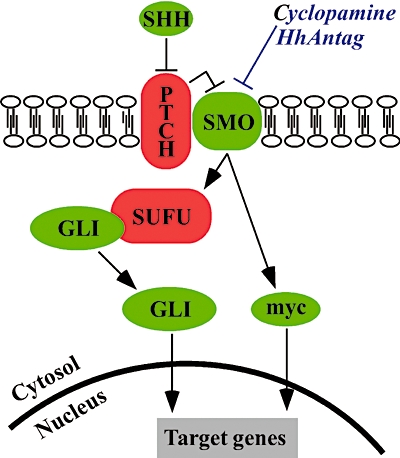
SHH signaling pathway components that have been employed in the production of genetically engineered mouse models. Oncogenes are shown in green, and tumor suppressors are shown in red. Examples of pharmaceutical agents are shown in italicized blue with their targets indicated.

Over the last decade and a half, numerous investigations have implicated sonic hedgehog (SHH) signaling in medulloblastoma pathogenesis (21, 67), and modulation of the SHH pathway has led to the vast majority of the medulloblastoma GEMMs currently available. A schematic of SHH signaling is shown in Figure 3. Briefly, the binding of SHH to its receptor patched (PTCH) removes the latter's inhibitory effects on the downstream effector smoothened (SMO). SMO then initiates signaling events leading to the release of the GLI family of transcription factors from inhibitory protein complexes that include suppressor of fused (SUFU). This process results in the eventual transcription of GLI target genes and the consequent physiologic effects, including cell proliferation in the right cellular context (39). The SHH pathway was first implicated in medulloblastoma when germline mutations in the *PTCH1* gene were found to be the cause of Gorlin's syndrome, a congenital condition characterized by increased incidence of basal-cell carcinoma, medulloblastoma and rhabdomyosarcoma (25). Since then, mutations in multiple components of the SHH signaling cascade have been identified in sporadic medulloblastoma, specifically inactivating mutations in PTCH1 and SUFU and activating mutations in SMO, together accounting for 15% of all cases (57, 59, 63, 75). Interestingly, medulloblastomas characterized by mutations in the SHH pathway tend to exhibit desmoplastic morphology.

Additional oncogenes and tumor suppressors have been linked to medulloblastoma pathogenesis. Germline mutations in p53 lead to Li-Fraumeni syndrome, which is characterized by increased incidence of a number of different tumor types, including medulloblastoma (46). Furthermore, p53 mutations have also been identified in sporadic variants of this tumor with poor clinical outcome (76). The oncogenes *N-MYC* and *C-MYC* are notably amplified in a subset of medulloblastoma that tends to demonstrate anaplastic features and aggressive biological behavior (1, 76). Finally, insulin-like growth factor-2 (IGF2) overexpression has been found in a portion of sporadic medulloblastoma, mainly of the desmoplastic subtype (58).

### Engineering and characteristics

As stated above, the plurality of medulloblastoma GEMMs generated to date derive from exogenous manipulation of the Shh pathway (Table 2). Mice hemizygous for *Ptch* (*Ptch*+/−) develop medulloblastoma at a relatively modest rate of 14%–19% by 10 months (23, 88, 98). A significant number of other tumors, most notably soft tissue sarcomas, also form in this GEMM (44, 88). Interestingly, the remaining Ptch allele in these mice appears to be functional in a majority of generated medulloblastomas, suggesting that mere haploinsufficiency increases Shh signaling enough to mediate tumorigenesis (54, 88, 98). Irradiating these mice during the early postnatal period dramatically increases tumor incidence—as high as 100% by 10 months—most likely due to loss of heterozygosity at the remaining *Ptch* locus, coupled with mutations in other relevant genes like *p53*(54, 55). Medulloblastoma frequency in the *Ptch*+/− model has also been improved through the use of more discrete genetic modifications. Complete loss of p53, for instance, greatly increases tumor incidence to 100% by 10–12 weeks (89), and functional deletion of the *Ptch* homologue *Ptch2* has similar, if more modest, effects (44). A related murine model employs hemizygous loss of *Sufu* in a *p53* null background (*Sufu*+/−/*p53*−/−), yielding medulloblastomas in 58% of mice over the course of 10 months (42). Lastly, a particularly robust GEMM has been generated using a constitutively active *Smo* allele (SmoA1) under the control of the granule neuron precursor-specific promoter ND2 (26, 28). Ninety four percent of homozygous SmoA1/SmoA1 mice develop medulloblastoma by 2 months of age, and these tumors frequently exhibit leptomeningeal spread, a common feature of the human disease (28). Furthermore, the localization of transgene expression to granule neuron precursors eliminates the occurrence of non-CNS pathology, perhaps simplifying the implementation of this model in preclinical studies. Indeed, a similar GEMM employing an activated Smo under a ubiquitously expressed inducible driver leads to widespread neoplastic lesions, including, but not limited to, medulloblastoma in 40% of cases (47).

**Table 2:** Murine models of medulloblastoma shown with underlying genetics, mechanism of engineering and incidence. Abbreviations: TG = transgenic, KO = knockout.

Genetics	Mechanism	Incidence	Reference
*Ptch*+/−	Conventional KO	14%–19% by 10 months	(23, 44, 98)
*Ptch*+/−	Conventional KO; irradiation	100% by 10 months	(54, 55)
*Ptch*+/−; p53−/−	Conventional KO	100% by 2–3 months	(89)
*Ptch*+/−*; Ptch2*±/−	Conventional KO	17% by 10 months	(44)
*Ptch*+/−	Conditional KO (GFAP-Cre, Math1-Cre)	100% by 1–3 months	(95)
*ND2-SmoA1*	TG	94% by 2 months	(26, 28)
*SmoM2*	Conditional TG (GFAP-Cre, Math1-Cre, Olig2-Cre, Tlx-Cre)	100% by 2–4 months*	(69)
*Sufu*+/−*; p53*−/−	Conventional KO	58% by 10 months	(42)
*Shh*	RCAS	9%–34% by 3 months	(7, 49, 60, 61)
*Shh; c-myc/Akt/IGF2*	RCAS	23%–48% by 3 months	(60, 61)
*Shh; n-myc*	RCAS	78% by 3 months	(7)
*Shh; Bcl-2*	RCAS	78% by 3 months	(49)
*Rb*±/−*; p53*−/−	Conditional KO (GFAP-Cre)	25%–100% by 2–7 months	(48)
*Ink4c*±/−*; p53*±/−	Conventional and conditional KO (Nestin-Cre); irradiation	20%–100% by 5 months	(81)
*Ptch*+/−*; Ink4c*±/−	Conventional KO	40%–50% by 9 months	(81)
*Lig4*−/−*; p53*−/−	Conventional KO	100% by 2 months	(43)
*Brca2*−/−*; p53*±/−	Conventional and conditional KO (Nestin-Cre)	72%–83% by 4–8 months	(17)
*Xrcc4*−/−*; p53*−/−	Conventional and conditional KO (Nestin-Cre)	100% by 6 months	(94)

* Estimated from reported average latency.

Two other recently developed model systems also minimize the incidence of non-CNS tumors by limiting causal genetic events to particular cell types. One series utilizes conditional *Ptch* knockouts paired with promoter-restricted Cre drivers (95). In this way, loss of *Ptch* is localized to either granule neuron precursors or more primitive cerebellar neural stem cells by using Math1-Cre or GFAP-Cre transgenes, respectively. The other group of GEMMs uses a similar strategy, employing Math1 and GFAP-driven Cre to instead direct the expression of an activated *Smo* allele. However, they also incorporate two additional drivers, Olig2-Cre and Tlx3-Cre, whose spatial expression pattern within the murine cerebellum differs from those of GFAP-Cre and Math1-Cre (69). In all cases, medulloblastomas with similar, if not identical, histological features, develop at robust levels—as high as 100% by 4 weeks of age. The variability of tumor incidence with regard to individual Cre-drivers does differ somewhat between the two model systems, most likely caused by the contrasting causative oncogenic events (ie, *Ptch* loss vs. forced *Smo* overexpression). Interestingly, the spatial distribution of the generated medulloblastomas corresponds to that of their transgenic driver, and, presumably, their cell of origin. This finding underscores the potential utility of these models in preclinical testing, despite their more complex and cumbersome genetics. The therapeutic targeting of different cell types within a heterogeneous tumor mass has become a major focus of translational cancer biology (see below). The ability to generate of an array of medulloblastomas from differing cells of origin provides and inviting substrate for investigations on this topic.

Medulloblastoma models have also been generated by retroviral gene transfer in a similar fashion to that used for the production glioma GEMMs. One group has used the RCAS/tv-a system to create a series of GEMMs driven by Shh expression (7, 49, 60, 61). Cerebellar application of RCAS-Shh alone to Ntv-a mice yields medulloblastomas at a rate of 9%–34% with a median latency of roughly 6–7 weeks (Figure 2). Co-injection of any one of a number of additional oncogenes, such as *c-myc*, *Akt*, *IGF2*, and *n-myc* enhances the strength of the phenotype, the greatest effects occurring with a combination of Shh and a stabilized *n-myc* mutant. This latter tumor type also exhibits an increased mitotic index reminiscent of the anaplasia associated with *myc* amplification in human medulloblastoma (7). RCAS-mediated expression of Shh, together with the antiapoptotic factor Bcl-2, also appears to increase the frequency of tumor formation (49). Decreased levels of apoptosis in this model have been confirmed.

Only a handful of medulloblastoma GEMMs not driven by Shh signaling have been developed to date (Table 2). Two such models utilize homozygous, for the most part, loss of p53 as the foundation of their genetic design. In one GEMM, this strategy is coupled with partial or complete loss of Rb in cerebellar granule neurons by way of a GFAP-Cre transgene and a floxed *Rb* allele (48). This process results in medulloblastoma formation in a majority of mice following a relatively long latency period—76 to 196 days—with the precise rate of incidence depending somewhat on the level of remaining Rb activity. In the other model system, medulloblastomas have been successfully generated by irradiating 5-day old mice harboring mutations in both *p53* and the tumor suppressor *Ink4c*, with an incidence between 20% and 100%, depending on the zygosity of both genes (81). The physiologic relevance of these models might provide some cause for question. While both germline and sporadic *p53* mutations have been associated with human medulloblastoma, a central role for the gene in the pathogenesis of the tumor is less certain; and defects in the Rb pathway are rarely seen. One might even expect gliomas to form in the first of these model systems, given its GFAP-dependent distribution of genetic lesions that seem more appropriate to that tumor type. However, none are reported (48). Regarding *Ink4c*, the same study that describes the GEMM reports methylation at the gene locus in 4 of 23 examined cases of human medulloblastoma. Additionally, *Ink4c* loss is applied to the standard *Ptch*+/− GEMM, leading to a modest enhancement of phenotype (∼45% incidence) (81).

Finally, another group of GEMMs has employed generalized genomic instability to effectively produce medulloblastoma in mice. In these models, p53 deficiency is typically paired with loss of crucial DNA repair enzymes, such as *Lig4*, *Xrcc4* and *Brca2*(17, 31, 43, 94). As an interesting side note, mutations in this latter gene have been associated with Fanconi's anemia, a systemic condition characterized by, among other things, a predisposition to develop medulloblastoma (52). The most effective of these models restrict their genetic defects to neuroglial progenitor cells using floxed alleles and nestin-Cre drivers. This results in a relatively high rate of medulloblastoma formation, with most mice succumbing within 120–180 days. Furthermore, genetic analysis of the generated tumors reveals mutations, as well as amplifications at genetic loci commonly affected in medulloblastoma, such as *Ptch*, *c-myc*, *n-myc* and *p53*(17, 94). Such findings support the use of these model systems in preclinical trials by highlighting their physiological relevance.

## CONSIDERATIONS FOR PRECLINICAL STUDIES USING BRAIN TUMOR GEMMS

### Pathway-targeted therapies

While none of the glioma or medulloblastoma GEMMs described in the preceding sections completely phenocopies their respective human conditions, their combined utility in the preclinical testing of therapeutic regimens remains obvious. Over the past two decades, rational drug design has led to numerous small molecule inhibitors targeting many of the oncogenic pathways involved in brain tumor pathogenesis (examples given in Figures 1 and 3), the same pathways modulated, either singly or in combination, in the design of glioma and medulloblastoma GEMMs. Consequently, these models constitute ideal *in vivo* systems in which to study the effects of particular drugs on their molecular targets and the consequences for tumorigenesis. Additionally, the systemic toxicity of individual compounds can be gauged, and strategies to follow clinical course formulated. Despite these possibilities, only a handful of such studies have been performed to date.

The effects of PTK787, a PDGFR and vascular-endothelial growth factor receptor (VEGFR) inhibitor, have been tested in the aforementioned RCAS-PDGF-mediated oligodendroglioma model (72, 80). A 70-day course of PTK787, given at 100 mg/kg/day, leads to lower-grade histology in treated tumors characterized by markedly decreased mitotic activity, although tumor vascularity appears unchanged (72). This GEMM has also been used to assess the *in vivo* impact of perifosine, an oral inhibitor of the AKT and RAS/MAPK pathways, both of which are known to be activated by the enhanced RTK signaling common in glioma (50). Mice treated with a combination of 100 mg/kg temozolomide and 30 mg/kg perifosine for 3–5 days exhibit tumors with a significantly decreased proliferation index. Furthermore, the combined effects of perifosine and temozolomide are greater than for either drug alone. This study demonstrates (i) how GEMMs can be used to test drugs targeting pathways that are not directly altered in the genetic design of the model (in this case the crucial signaling networks are downstream of the exogenous oncogenic stimulus); and (ii) the utility of GEMMs in the analysis of multiple drugs or therapeutic modalities in combination.

Similar studies have been performed on glioma GEMMs exhibiting more astrocytic histology. The previously described RCAS-kRas/RCAS-Akt-driven astrocytoma model has been used to evaluate CCI-779, an inhibitor of mTOR (36). Treated tumors harbor large areas of necrosis and apoptosis, and, interestingly, surrounding viable tissue exhibits a more oligodendroglial morphology. These findings not only highlight the therapeutic potential of CCI-779 and its related compounds, but also underscore the importance of mTOR signaling both for tumor survival and the maintenance of astrocytic character. This study also yields relevant dosing information, with the effective daily regimen of 40 mg/kg far exceeding the 0.1 mg/kg found to be active in xenografts, illustrating the importance of an intact blood–brain barrier for the proper *in vivo* analysis of potential brain cancer therapies. The impact of Ras pathway inhibition has also been assessed in the homozygous *Nf1*-deleted OPG model mentioned earlier. In these mice, the mTOR inhibitor rapamycin appears to decrease both tumor cell proliferation and tumor size in a dose-dependent fashion (29). The study incorporates temozolomide treatment as well, although no enhanced effect is seen when combining the two drugs.

Medulloblastoma models have also been used effectively in the preclinical testing of targeted therapeutics. The recent finding that SHH pathway activity is downregulated in medulloblastoma cells once they are placed in tissue culture further emphasizes the importance of *in vivo* systems in these types of investigations (6, 68). *Ptch*+/−/*p53*−/− mice treated with HhAntag, an inhibitor of SHH signaling, exhibit complete tumor eradication at a dose of 100 mg/kg/day (66). Additionally, mice maintained on HhAntag remain medulloblastoma-free for as many as 147 days. These dramatic effects, however, have been somewhat tempered by subsequent studies identifying bone defects in young mice after even transient exposure to this drug (40).

### Pharmacology by way of genetic engineering

GEMMs need not be treated directly with drugs to successfully inform subsequent clinical trials. Indeed, the potential effects of targeting vital oncogenic pathways in brain tumors can be modeled simply by manipulating the underlying genetics of relevant GEMMs. In this way, the requirement of a tumor for a particular oncogenic stimulus can be assessed, even in the absence of a test compound. This approach has been applied for glioma using an RCAS-kRas/RCAS-Akt-driven astrocytoma model, in which kRas expression is dependent on the administration of doxycycline (35). Addition of doxycycline after tumor formation leads to complete loss of kRas expression and dramatic tumor regression and subsequent withdrawal of tetracycline restores kRas expression, resulting in tumor recurrence within 3 weeks. These findings indicate a requirement for Ras signaling in the maintenance astrocytoma, bolstering the case for inhibition of this pathway in antiglioma therapies.

### Radiotherapy

Ionizing radiation constitutes a major therapeutic modality in the treatment of both glioma and medulloblastoma, although much of the biology underlying its effects and the mechanisms of tumor cell resistance remain unclear. The high fidelity to which GEMMs recapitulate both the genetics and the characteristic tumor–stroma interactions of brain cancer would seem to make them ideal systems for the refinement of more effective radiotherapy regimens, especially in combination with synergistic drugs. Nevertheless, this avenue of translational research remains largely unexplored. An investigation into the irradiation of medulloblastoma using the RCAS-Shh-based model system has recently been reported (27). Radiotherapy is found to induce widespread, p53-mediated, apoptotic cell death in the tumor bulk, which is largely absent in a *p53* null background. Furthermore, a subset of radioresistant stem-like tumor cells characterized by their perivascular distribution (9) appear to activate PI3K/Akt/mTOR signaling following irradiation, undergo brief cell-cycle arrest and then gradually begin dividing again, presumably leading to disease recurrence. Intriguingly, combined radiation and treatment with of the AKT pathway inhibitor perifosine appears to significantly decrease survival in this cellular subpopulation. These findings not only provide *in vivo* insight into the biology of irradiated tumors, but also uncover a promising therapeutic strategy for radiosensitization in medulloblastoma. On a different level, this study also indicates that p53 loss matters greatly with regard to radiotherapy, and that *p53*−/−tumors will not behave in the same fashion as the majority of human medulloblastomas that are *p53*-intact.

### Cancer stem cell biology

The cellular heterogeneity exhibited by some brain tumor GEMMs, as highlighted in the preceding paragraph, offers a distinct advantage when studying the impact of test therapies on important subpopulations of cells like CSCs. As we have seen, the causative genetic or molecular events in GEMMs are frequently targeted to progenitor cells or other similar groups with stem-like character (Tables 1 and 2). Furthermore, tumors in some models even appear to localize at their earliest stages to brain regions rich in multipotent stem cells (96). The means to identify CSCs within tumor masses are also improving steadily, as evidenced by identification of the brain CSC marker CD133 (73). Recently, one group has taken advantage of the singular ability of stem cells to efflux drugs, as well as the fluorescent Hoechst dye, to isolate CSCs from a PDGF-driven glioma model by fluorescent-activated cell sorting (Bleau, AM and Holland, EC, unpub. obs.). By obtaining a relatively pure population of stem-like cells, they are able to demonstrate the importance of the Akt pathway in the maintenance of CSC characteristics, in particular the resistance to alkylating agents like temozolomide. Additional studies like this one should further clarify mechanisms for the effective targeting of this crucial class of tumor cells.

### *In vivo* imaging

Improvements in imaging technology have further enhanced the promise of preclinical testing in brain tumor GEMMs. Magnetic resonance imaging (MRI) has already been successfully employed in the analysis of drug effects on brain tumor models of both oligodendroglial and astrocytic lineage (36, 72). In these studies, high-grade lesions were identified by MRI and followed serially during treatment with observed decreases in contrast enhancement. Drug efficacy was then confirmed histologically. The more recent development of genetically engineered bioluminescent reporter mice has brought with it the ability to query distinct molecular pathways and signaling networks and assess their response to therapeutic intervention. One such system utilizes an E2F1-luciferase transgene, expressed primarily in dividing cells, that mediates the release of light in the presence of the bioluminescent compound luciferin. Consequently, groups of rapidly multiplying cells in these mice, such as tumors, release sufficient light so as to be detectable by a sensitive luminometer, even when transmitted through skin, soft tissue and bone. This strategy, in the context of a PDGF-driven glioma GEMM, has been used to successfully monitor the effects of pharmacologic PDGFR inhibition on tumor growth (80). Other reporter mice have also been generated, including a GLI-response luciferase transgenic that demonstrates Shh signaling (6). The creation of these additional bioluminescent strains for the analysis of molecular pathways implicated in brain tumor biology should greatly streamline the process of testing targeted therapies in murine model systems.

## CONCLUDING REMARKS

As advances in cancer biology continue to reveal the molecular mechanisms underlying brain tumor pathogenesis, the success with which such discoveries are translated into effective therapies has come to depend on the ability of the scientific community to rapidly screen potential therapies in appropriate *in vivo* systems. Well-designed drug tests in murine models of brain cancer should not only serve to rapidly isolate promising therapeutic strategies, but should also better inform subsequent trials in human subjects, and, in doing so, both lower their cost and increase their efficacy. This review has attempted to briefly overview the currently available glioma and medulloblastoma GEMMs, and their potential for applications in preclinical testing. In many ways, these murine model systems remain a largely untapped resource (65), as evidenced by the relatively small number of studies successfully employing GEMMs in preclinical trials to date. More extensive use of brain tumor GEMMs in this kind of translational research will hopefully underscore their utility as effective disease models and facilitate the development of the next generation of molecularly targeted therapeutics.
